# The multivisceral landscape of colorectal cancer metastasis: implications for targeted therapies

**DOI:** 10.1172/JCI178331

**Published:** 2024-03-01

**Authors:** Dominik Wolf, Stefan Salcher, Andreas Pircher

**Affiliations:** 1 Internal Medicine V, Department of Hematology and Oncology, Comprehensive Cancer Center Innsbruck (CCCI), Medical University of Innsbruck (MUI), Innsbruck, Austria.; 2 Tyrolean Cancer Research Institute (TKFI), Innsbruck, Austria.

## Abstract

Colorectal cancer (CRC) is among the most common cancer types and the second deadliest malignancy for both sexes. Metastatic disease poses substantial therapeutic challenges, and peritoneal spread, in particular, reduces quality of life and has a dismal outcome. In this issue of the *JCI*, Berlin and authors have made considerable advancements in understanding the cellular and molecular composition of multivisceral CRC metastasis in a sophisticated murine orthotopic organoid model and in humans. The study provides unprecedented insights into the complex biology of the disease and points toward the development of compartmentalized immune-therapeutic strategies.

## Peritoneal carcinomatosis tissue

**Figure 1:**
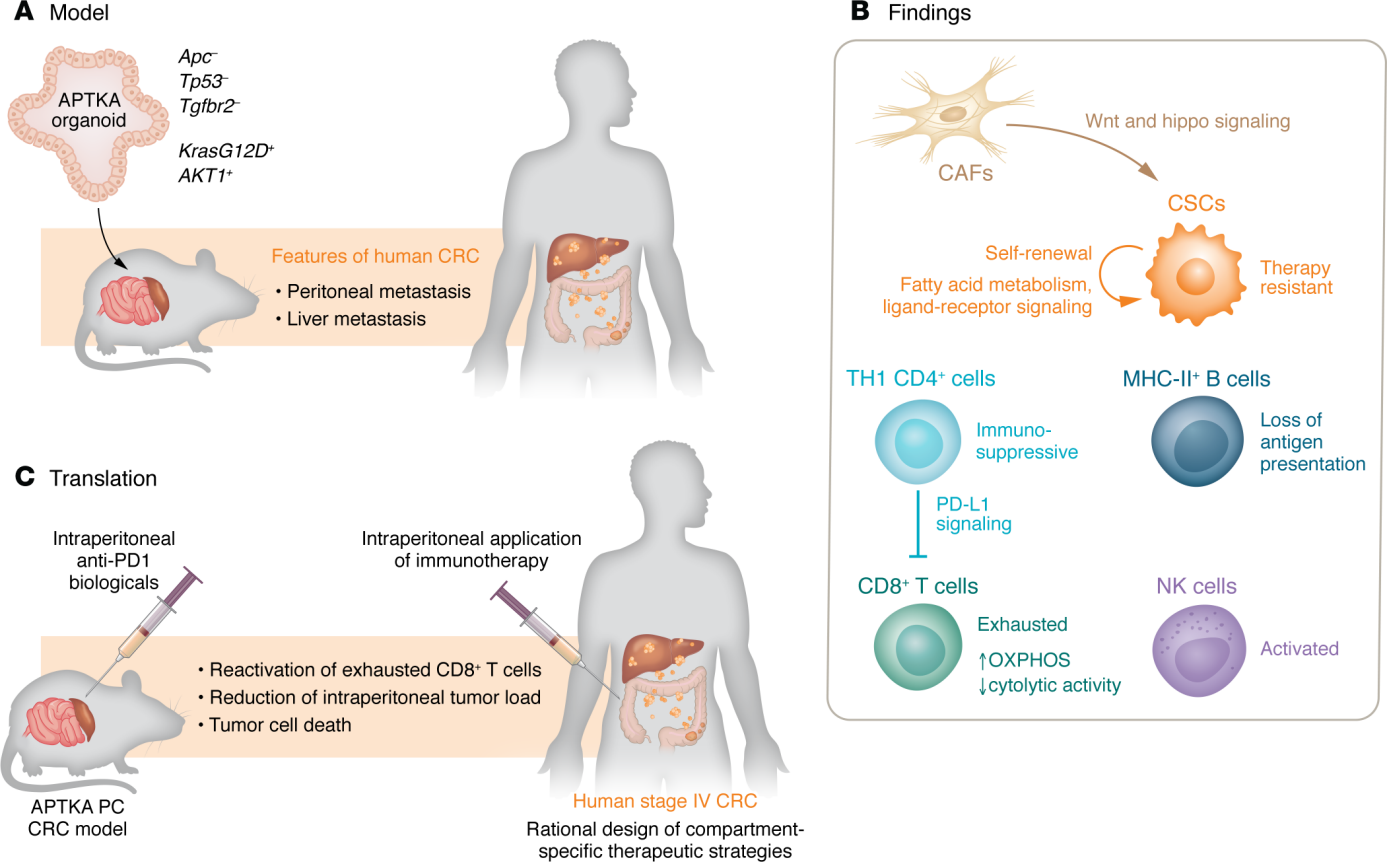
An orthotopic, organoid-driven immunocompetent mouse model reveals therapy-resistant cancer stem cells and the value of compartment-specific therapy. (**A**) Berlin et al. developed the APTKA organoid CRC model. Notably, the multivisceral landscape of CRC metastasis driven by the APTKA organoid model mimics human stage IV CRC. (**B**) Single-cell deconvolution of the CRC metastatic sites identified CSCs as important regulators of PC. CAFs interact with CSC via important CSC-inducing pathways. Next, an immunosuppressive TME becomes generated by the presence of exhausted CD8^+^ T cells, immunosuppressive Th1 CD4^+^ cells, and B cells with loss of antigen-presentation machinery. In contrast, NK cells activate and shift their metabolic status toward OXPHOS. (**C**) Mice with APTKA PC CRC responded to a PC-specific therapeutic approach. The findings suggest treatment of anti-PD1 biologicals by intraperitoneal application may reactivate exhausted CD8^+^ T cells and increase tumor cell death of intraperitoneal CRC in patients.

Colorectal cancer (CRC) is the third most common cancer diagnosis and second deadliest malignancy for both sexes combined, posing substantial challenges, especially in its advanced stages, characterized by metastasis to distant organs (1). In this issue of the *JCI*, Berlin and researchers utilized an orthotopic, organoid-driven immunocompetent mouse model that recapitulates the features of human disease with peritoneal and liver metastases (Figure 1) (2). Unlike in previous studies that focus on specific tumor cell populations or limited comparisons between two tumor sites, Berlin et al. comprehensively mapped the location-dependent cellular dynamics during metastasis. Leveraging the power of single-cell RNA sequencing (scRNA-Seq), the study enabled a granular examination of the tumor microenvironment (TME), revealing distinct intercellular communication networks and metabolic changes that occur during the process of metastasis to different locations.

One of the most important findings of Berlin et al. was the identification of cancer stem cells (CSCs) as dominant players in peritoneal carcinomatosis (PC) tissue. PC is a common feature of CRC and poses challenges to oncologists and patients. Clinically, PC is often linked to pain and ascites, which markedly reduce patients’ quality of life and result in a very bad prognosis, because PC is hard to tackle by systemic therapeutics (3). In the model provided by Berlin and colleagues, the identified CSCs created a self-promoting niche supporting their lodging through defined and CSC niche–specific signaling mechanisms, particularly the intercellular ligand-receptor signaling pathways (i.e., collagen, osteopontin, fibronectin, and thrombospondin signaling from CSCs in PC tissue) and exhibited an enhanced reliance on fatty acid metabolism (2). This observation aligns with previous studies highlighting the crucial role of fatty acid metabolism in maintaining stemness, self-renewal abilities, and therapy resistance in CSCs (4). The study further revealed the supporting role of cancer-associated fibroblasts (CAFs) in this niche, leading to increased Wnt and Hippo signaling in CSCs, which supports recent data proving that stromal fibroblasts regulate colonic epithelia differentiation (2, 5). The implications of these findings extend beyond CRC, resonating with existing research on the diverse subtypes of CAFs identified in various cancer entities (6).

## The immune landscape of metastatic CRCs

Berlin et al. (2) also investigated the immune landscape of metastatic CRC, uncovering nuanced alterations in the functionality of immune cells. Notably, adaptive antitumoral immune cells, such as cytotoxic CD8^+^ T cells, exhibited a loss of cytolytic activity and adopted an exhausted phenotype in the metastatic TME. This phenotype was accompanied by an upregulation of oxidative phosphorylation (OXPHOS) metabolism (7). Paradoxically, other cytotoxic-acting cells, such as natural killer (NK) cells, gained increased activity in liver and peritoneal metastases. Metabolic reprogramming toward OXPHOS and the simultaneous activation of NK cells have been previously observed in various diseases (8, 9), and this study provides critical insights into their potential role during CRC metastasis.

Berlin et al. (2) also distinguished intermetastatic Th1 CD4^+^ T cell function, showcasing the predominance of immunosuppressive PD-L1 signaling in peritoneal carcinomatosis toward CD8^+^ effector cells. This trend was accompanied by a loss of B cell–dependent MHC-II signaling, potentially indicating that within peritoneal metastases, MHC-II^+^ B cells possess a disturbed antigen-presenting function. Interestingly, the recently published multiplexed 3D CRC atlas shows similar results and identified the highly proliferative nature of tumor cells in deep invasive margins, accompanied by fewer adjacent immune cells. Notably, PD-L1–expressing myeloid cells surpass PD-L1–expressing tumor cells in abundance across 17 examined CRCs in the atlas. High-resolution imaging emphasizes the frequent contacts between myeloid cells and PD1^+^ T cells, highlighting dendritic cells as primary contributors to immunosuppressive PD-L1, aligning with their known tolerization roles (10).

## Clinical implications

Translating these findings into a clinical context, Berlin and colleagues demonstrated a strong concordance between the proposed orthotopic murine model and human multilocular metastasis in CRC. Bulk proteomic and transcriptomic analyses indicated a congruent deconvolution of the configuration of murine and human liver metastases as well as PC tissue. The study’s reliance on tumor organoids, rather than permanent cell lines, underscores its commitment to reproducing the mutational burden and architecture of the original tumor, making it a more faithful representation of the disease in vivo (2).

The methodological innovation of the study also sets it apart from previous metastatic CRC mouse models. Unlike strategies relying on direct intraperitoneal or intrasplenic injections of tumor cell lines, which do not sufficiently mimic the physiological route of metastasis, Berlin et al. (2) utilized surgically implanted organoids deficient for *Apc*, *Tp53*, and *Tgfbr2* carrying a *Kras*G12D mutation and an activated/myristoylated isoform of AKT1 (termed APTKA). The APTKA organoids were placed into the subserosa of the cecum of immunocompetent mice. While the infiltrative direction in this model was inverted compared with the physiological condition of CRC tumorigenesis, the study demonstrated a transluminal epithelial barrier breach as early as seven days after organoid implantation. This method allowed for the exposure of tumor cells to intestinal stimuli similar to what is seen in human CRC, offering a more clinically relevant model for studying multivisceral CRC metastasis (2).

Most relevant to potential clinical translation, Berlin and researchers proposed a PC-specific therapeutic approach based on their findings. In a proof-of-concept experiment, murine APTKA PC CRC was successfully treated by intraperitoneal application of anti-PD1 biologicals, which reactivated exhausted CD8^+^ T cells and increased tumor cell death of intraperitoneal tumors (2). This outcome not only underscores the potential of checkpoint inhibition as a therapeutic strategy, but also emphasizes the necessity for location-specific development of therapeutic interventions (7). The observations made in Berlin et al. (2) reinforce the idea that a one-size-fits-all approach to CRC treatment is inadequate. The study draws attention to the specific adaptations of tumor cells and the TME to local environments during disease progression, emphasizing the need for targeted and location-specific therapeutic interventions. For instance, the study challenges the efficacy of intraperitoneal and systemic application of chemotherapeutic agents in peritoneal carcinomatosis, suggesting that these traditional approaches may have limited efficacy in certain contexts (2). The use of hyperthermic intraperitoneal chemotherapy (HIPEC) in PC is one such compartmentalized approach tested in selected patients with isolated PC upon cytoreductive surgery (CRS) (11). However, the most recent data of the PRODIGE7 trial do not support an additional OS benefit of CRS in conjunction with HIPEC versus CRS alone (12), pinpointing the unmet need for more targeted and rational therapeutic approaches in PC.

In summary, Berlin et al. (2) present a comprehensive and detailed cellular and molecular landscape of multivisceral CRC, shedding light on the intricate processes of metastatic adaptations to local environments. The findings not only contribute to our understanding of the complex biology of CRC, but also identify exhausted effector CD8^+^ T cells as potential PC-specific therapeutic targets. The implications of this research extend beyond CRC, providing a paradigm for the nuanced exploration of metastatic landscapes in various cancers and paving the way for a new era of precision medicine in oncology.

## Version 1 03/01/2024

Electronic publication

## References

[1] Torre LA (2016). Global cancer incidence and mortality rates and trends--an update. Cancer Epidemiol Biomarkers Prev.

[2] Berlin C (2024). Single-cell deconvolution reveals high lineage- and location-dependent heterogeneity in mesenchymal multivisceral stage 4 colorectal cancer. J Clin Invest.

[3] Tseng J (2017). Under-representation of peritoneal metastases in published clinical trials of metastatic colorectal cancer. Lancet Oncol.

[4] Yi M (2018). Emerging role of lipid metabolism alterations in cancer stem cells. J Exp Clin Cancer Res.

[5] Qin X (2023). An oncogenic phenoscape of colonic stem cell polarization. Cell.

[6] Caligiuri G, Tuveson DA (2023). Activated fibroblasts in cancer: Perspectives and challenges. Cancer Cell.

[7] He K (2023). Metastasis organotropism in colorectal cancer: advancing toward innovative therapies. J Transl Med.

[8] Assmann N (2017). Srebp-controlled glucose metabolism is essential for NK cell functional responses. Nat Immunol.

[9] Donnelly RP (2014). mTORC1-dependent metabolic reprogramming is a prerequisite for NK cell effector function. J Immunol.

[10] Lin JR (2023). Multiplexed 3D atlas of state transitions and immune interaction in colorectal cancer. Cell.

[11] Verwaal VJ (2003). Randomized trial of cytoreduction and hyperthermic intraperitoneal chemotherapy versus systemic chemotherapy and palliative surgery in patients with peritoneal carcinomatosis of colorectal cancer. J Clin Oncol.

[12] Quénet F (2021). Cytoreductive surgery plus hyperthermic intraperitoneal chemotherapy versus cytoreductive surgery alone for colorectal peritoneal metastases (PRODIGE 7): a multicentre, randomised, open-label, phase 3 trial. Lancet Oncol.

